# A Hybrid of Random Forests and Generalized Path Analysis: A Causal Modeling of Crashes in 52,524 Suburban Areas

**DOI:** 10.34172/jrhs.2023.116

**Published:** 2023-06-29

**Authors:** Fatemeh Jahanjoo, Homayoun Sadeghi-Bazargani, Mohammad Ali Mansournia, Seyyed Teymoor Hosseini, Mohammad Asghari-Jafarabadi

**Affiliations:** ^1^Road Traffic Injury Research Center, Tabriz University of Medical Sciences, Tabriz, Iran; ^2^Department of Epidemiology and Biostatistics, School of Public Health, Tehran University of Medical Sciences, Tehran, Iran; ^3^Department of Engineering Traffic and Transportation, Faculty of the Traffic, Tehran University, Tehran, Iran; ^4^Cabrini Research, Cabrini Health, Malvern, VIC 3144, Australia; ^5^Biostatistics Unit, School of Public Health and Preventative Medicine, Faculty of Medicine, Nursing and Health Sciences, Monash University, Melbourne, VIC 3004, Australia; ^6^Department of Psychiatry, School of Clinical Sciences, Faculty of Medicine, Nursing and Health Sciences, Monash University, Clayton, VIC 3168, Australia

**Keywords:** Accident, Traffic accidents, Causal effect, Regularization algorithm, Generalized path analysis

## Abstract

**Background:** Determining suburban area crashes’ risk factors may allow for early and operative safety measures to find the main risk factors and moderating effects of crashes. Therefore, this paper has focused on a causal modeling framework.

**Study Design:** A cross-sectional study.

**Methods:** In this study, 52524 suburban crashes were investigated from 2015 to 2016. The hybrid-random-forest-generalized-path-analysis technique (HRF-gPath) was used to extract the main variables and identify mediators and moderators.

**Results:** This study analyzed 42 explanatory variables using a RF model, and it was found that collision type, distinct, driver misconduct, speed, license, prior cause, plaque description, vehicle maneuver, vehicle type, lighting, passenger presence, seatbelt use, and land use were significant factors. Further analysis using g-Path demonstrated the mediating and predicting roles of collision type, vehicle type, seatbelt use, and driver misconduct. The modified model fitted the data well, with statistical significance ( $\chi_{30}^{2}$ = 81.29, *P*<0.001) and high values for comparative-fit-index and Tucker-Lewis-index exceeding 0.9, as well as a low root-mean-square-error-of-approximation of 0.031 (90% confidence interval: 0.030-0.032).

**Conclusion:** The results of our study identified several significant variables, including collision type, vehicle type, seatbelt use, and driver misconduct, which played mediating and predicting roles. These findings provide valuable insights into the complex factors that contribute to collisions via a theoretical framework and can inform efforts to reduce their occurrence in the future.

## Background

 Road traffic accidents (RTCs), as the cause of about 1.35 million deaths and 50 million serious injuries worldwide, represent a severe social and economic problem. In addition, it considers approximately 3% of the gross domestic product in 2018.^1,2^ Although all RTCs are problematic, research shows that fatality rates in suburban regions are greater.^3,4^ Suburban roadways play a transitional high-speed roadway role in connecting low-speed urban roads with high-speed rural highways. Notably, suburban areas have the properties of both urban (i.e., use of gutter and curb for drainage) and rural (i.e., high-pace) roadways. Consequently, enhancement in road safety seems to be refuted if these commutating areas are not taken into consideration. Therefore, to reduce road accidents and its consequence on road traffic, analyzing the characteristics of suburban crashes separately and providing the corresponding statistical model in full detail are of utmost importance.^5^

 Traditional statistical modeling, including regression models and linear approaches, has consistently been implemented in crash severity analysis.^6,7^ However, these models have to fulfill several assumptions about the fundamental structure of data and the structure of the relationships between independent and dependent variables. If the assumptions are violated, biased estimations and improper inferences can be obtained.^8^ Machine learning techniques as applied statistical methods have been considerably utilized in data analysis. These techniques do not contain pre-defined relationships between study variables, and the prediction is available without needing to understand essential mechanisms. These methods are currently successful due to the development of computational power.^6,9^

 Additionally, even though large population studies are routinely used to estimate the effect of predictors in actual situations, they are subject to confounding bias due to the lack of randomization. Hence, methods from the causal inference framework could be investigated as a strategy for developing sound and relevant science. Moreover, there is always difficulty with the number of variables that must be entered into the conceptual diagram of causal modeling, particularly in traffic studies with many risk factors. First, relying solely on substantive knowledge makes it challenging to detect true confounders. Second, neglecting a true confounder could result in biased conclusions, while accounting for non-confounders could raise variance.^10,11^

 Based on the literature in various disciplines, random forests (RF) as machine learning techniques and path analysis as a causal approach were revealed to be a good approach for road trafﬁc crash injury severity prediction.^12,13^ The RF proves to be a reliable algorithm for feature selection, even if the number of features is high. It has proven itself to be reliable, robust, and efficient. Furthermore, it outperforms other black-box algorithms as it is trained by a bootstrap aggregating (bagging) algorithm. This not only enhances the stability and accuracy of individual trees but also reduces variance and prevents over-fitting. The RF is also known for its interpretable model by producing a set of boosted if-then rules.^14,15^ Path analysis is a useful statistical tool for investigating the causal relationships between variables. It combines bivariate and multi-variable linear regression to examine the causal relations among the variables in model.^16^ This method can accurately determine the influence and significance of the relationship between various variables.^17^ In this paper, a hybrid random forest generalized path analysis (HRF-gPath) method was proposed to maintain sufficient number and efficient variables in the causal model of suburban area crashes. Beyond the methodological novelty proposed in this paper, combining these methods would lead to optimal feature selection and provide a powerful causal approach for a better conclusion. The results of this study can prepare guidelines and provide information for specialists to decide on the crucial risk factors of traffic crashes in suburban areas based on scientific evidence.

## Methods

###  Study design 

 This cross-sectional study analyzed the information on suburban crashes recorded in Integrated Road Traffic Injury Registry System (IRTIRS)^18^ from March 2015 to March 2016. The IRTIRS development as a national research study was started in 2017. The World Health Organization, the Iranian Ministry of Health, the Iranian Traffic Police, and the Iranian Forensic Medicine Organization are in charge of this multi-method study. In collaboration with other interested organizations, the Ministry of Health and Medical Education and the Road Traffic Injury Research Center affiliated with Tabriz University of Medical Sciences have taken steps to develop the information registration system to create an integrated system for data collection.

###  Ethics approval and consent to participate 

 The study was conducted following the Declaration of Helsinki and approved by the Institutional Review Board (#1396.465) and the Ethics Committee (#IR.TBZMED.REC.1398.1244) of Tabriz University of Medical Sciences, Iran. Participation in the study was voluntary for everyone, and participants’ privacy was respected. The participants were assured that their personal information would remain confidential and not be disclosed. Informed consent was obtained from both the adult participants and the parent(s)/guardian(s) of all under-16s; furthermore, informed consent was obtained from legal guardians or next of kin for illiterate participants. All methods were performed following the relevant guidelines and regulations. Finally, informed consent was obtained from all individual participants included in the study.

###  Data collection and study variables

 The scene of the crash-, vehicle-, and driver-based information was collected in the most critical provinces in Iran, which are either capital city destinations, tourism destinations, or free zone areas. Crash-based information included passenger presence, pedestrian presence, crash day, crash type, time, lighting status, weather, zone type, intersection control, line making, road material, land use, crash mechanism, view obstacle, and crash position. Other crash-related information were road surface, geometric design, vehicle factor, human factor, cause of the accident, collision type, distinct, road shoulder, road defect, permitted speed, and road repairing status. Moreover, vehicle-based information contained vehicle safety equipment, type, color, life, maneuver, plaque description, moving direction, and maneuver. Eventually, driver-based information included age, gender, education, job, driving license, seat belt usage, judiciary cause, and misconduct. This study divided the district into three categories, including tourist destinations, capital destinations, and free zones. As the final issue, the crash severity has three categories: property damage, injury, and fatality. Based on the study purpose, severity data were categorized into two distinct categories, including (1) damage or injury as a non-fatal crash (Y = 0) and (2) fatality as a fatal crash (Y = 1). There were 2,399 (4.57%) fatal crashes out of 52,524 suburban crashes. Overall, the information related to 42 explanatory variables was recorded, the details of which are presented in Table 1.

**Table 1:** Explanatory variables description in suburban area crashes based on the Iranian Integrated Road Traffic Injury Registry System (2015-2016)

**Variable **	**Total crashes**		**Fatal crashes**	
	**Number**	**Percent**	**Number**	**Percent**
Passenger presence	7643	14.55	1077	14.09
Pedestrian presence	1612	3.07	172	10.67
Crash day (Weekend)	16 002	30.47	802	5.01
Lightning				
Day	35 790	68.14	1350	3.77
Night	14 482	27.57	900	6.21
Twilight/dawn	2252	4.29	149	6.62
Weather				
Clear/cloudy	50 601	96.34	2327	4.60
Foggy/stormy/dusty	211	0.40	4	1.90
Rainy	1400	2.67	65	4.64
Snowy	312	0.59	3	0.96
Zone type				
Smooth	49 690	94.60	2219	4.47
Rough	778	1.48	58	7.46
Mountainous	2056	3.91	122	5.93
Existing intersection control	43 147	82.15	1905	4.42
Road lane line marking				
Broken line	3831	7.29	331	8.64
No line	124	0.24	5	4.03
Solid line	13 840	26.35	841	6.08
Double solid line	38 684	73.65	1558	4.03
Road material (Asphalt)	315	0.60	29	9.21
Land use				
Residential	9696	18.46	268	2.76
Nonresidential	34 195	65.10	1755	5.13
Other uni-purpose areas	7386	14.06	318	4.31
Multipurpose area	1247	2.37	58	4.65
Crash mechanism				
Single-vehicle crashes	13 425	25.56	691	5.15
Multiple-vehicle crashes	29 587	56.33	1087	3.67
Involving vulnerable road user crashes	9512	18.11	621	6.53
Existing view obstacle	1291	2.46	72	5.58
Crash position in riding lane	46 823	89.15	1959	4.18
Dry road surface	50 302	95.77	2303	4.58
Curved geometric design	6236	11.87	469	7.52
Existing vehicle factor	480	0.91	29	6.04
Existing human factor	31 166	59.34	1639	5.26
First cause				
More training	36 496	69.48	1664	4.56
Irresponsibility	10 327	19.66	237	2.29
More training and irresponsibility	5430	10.34	445	8.20
Failure of state organs	61	0.12	7	11.48
Multiple factors	210	0.40	46	21.90
Prior cause				
Hasty driving	20 478	38.99	863	4.21
Lack of attention to driving	24 238	46.15	904	3.73
Hasty driving and lack of attention to driving	3109	5.92	183	5.89
Lacked skill	2155	4.10	163	7.56
Other	2544	4.84	286	11.24
Direct cause				
Regulation	44 070	83.90	1876	4.26
Delay in sighting	4426	8.43	299	6.76
Overspending	3631	6.91	209	5.76
Escaping crash in a wrong way or multiple factors	397	0.76	15	3.78
Collision type				
Rear-end collisions	7958	15.15	912	11.46
T-bone collision	22 694	43.21	732	3.23
Head-on collision	15 624	29.75	584	3.74
Side-swipe collision	4333	8.25	93	2.15
Fixed-object collision	1915	3.65	78	4.07
Distinct				
Tourism destination	37 116	70.66	2065	22.55
Capital city destination	12 661	24.11	238	61.88
Free zone	2747	5.23	96	3.49
Road shoulder				
Unpaved	20 181	38.42	643	3.19
Soil	19 158	36.47	1007	5.26
Asphalt	13 185	25.10	749	5.68
Road defect				
No	47 458	90.35	1988	4.19
Pavement/lightning defects	1643	3.13	106	6.45
Signs defects	1360	2.59	119	8.75
Geometric defects	593	1.13	40	6.75
Multiple defects	1470	2.80	146	9.93
Permitted speed (km/h)				
≤ 30	3133	5.96	95	3.03
30-50	14 043	26.74	267	1.90
50-60	11 070	21.08	258	2.33
60-80	7754	14.76	303	3.91
80-95	6883	13.10	702	10.20
95-110	7442	14.17	609	8.18
110-120	2199	4.19	165	7.50
Road repairing	1050	2.00	53	5.05
Having vehicle safety equipment	6175	11.76	318	5.15
Vehicle color (High risk)	19 846	37.78	954	4.81
Vehicle life (Year)				
< 5 years	11 585	22.06	609	5.26
5-9	26 362	50.19	972	3.69
10-14	11 516	21.93	534	4.64
≥ 15	3061	5.83	284	9.28
State vehicle plaque description	15 217	28.97	861	5.66
Vehicle moving direction (Cardinal)	331	0.63	14	4.23
Vehicle maneuver				
Moving forward	49 989	95.17	2251	4.50
Turning	1806	3.44	58	3.21
Overtaking	111	0.21	20	18.02
Moving backward	209	0.40	7	3.35
Stopping on the road	127	0.24	12	9.45
Other	282	0.54	51	18.09
Driver being at fault	34 032	64.79	1660	4.88
Driver gender (Male)	50 188	95.55	2331	4.64
Driver education				
Illiterate	1418	2.70	69	4.87
Primary	3996	7.61	148	3.70
Nonacademic	43 115	82.09	2062	4.78
Academic	3995	7.61	120	3.00
Driver job				
Jobs with high economic status	46 689	88.89	2142	4.59
Jobs with middle economic status	3454	6.58	176	5.10
Jobs with low economic status	2381	4.53	81	3.40
Driver age				
Child	477	0.91	24	5.03
Adult	49 497	94.24	2246	4.54
Elderly	2550	4.85	129	5.06
Type of driving license				
Class A	8434	16.06	336	3.98
Class B	16 015	30.49	408	2.55
Class C	26 422	50.30	1580	5.98
Motorcycle	458	0.87	3	0.66
No license	1195	2.28	72	6.03
Driver does not wear a seatbelt	20 463	38.96	1150	5.62
Driving carelessly	1415	2.69	181	12.79
Driver misconduct				
Spiral movement	27 169	51.73	139	0.51
Over speeding	21 313	40.58	1630	7.65
Other	4042	7.70	630	15.59

###  Statistical analysis

 Statistical data were analyzed using STATA (Release 17: 2021, StataCorp LCC, College Station, Texas 77845-4512 USA), TIBCO STATISTICA (Release 13.5.0.17: 2018, Statsoft, inc. USA), and MPlus (Release 7.4: 2015, Los Angeles, CA: Muthén & Muthén). The proposed hybrid model initiates with the RF classifier for variable selection, followed by generalized path analysis to conduct causal modeling. In the first step of the proposed HRF-gPath model, the RF classifier efficiently reduces less important variables and enhances the proposed model’s generalization capabilities. The RF is a supervised machine learning technique introduced by Breiman’s^19^ and focuses on the “decision tree” approach implemented in the classification and regression tree methodology. The decision tree is considered a technique for classifying data that are divided into groups based on the value of a particular variable. Then, it repeats this division such that each data group comprises objective variables in the same category. In this method, the basis of most decisions is classification. In addition, the importance of each variable and the contribution of each variable in data classification can be determined by the created decision trees. This study used classification algorithms to predict a categorical dependent variable. The risk was calculated as the proportion of cases incorrectly classified by the trees. The Gini index (GI) was employed to reduce the node impurity. Our optimal model was trained to have a GI around 0.1. To control all key aspects of the estimation procedure and model parameters, including the complexity of the trees fitted to the data, the maximum number of trees in the forest was set to 100. Additionally, to control how to stop the algorithm when achieving satisfactory results, the maximum number of leaves was set to 10.^19^ The data were randomly split into training and test sets so that the training set consisted of 80% of the full data set, while the test set comprised the remaining 20%. The training set was utilized to fit (train) the model. The test set was used to evaluate the fitted RF performance and determine whether it is overfitting. The research team took the mid-point of 0.5 as the cutoff point for deciding on the feature selection criterion and introducing it to gPath analysis.

 To maximize the advantages of the algorithm in this hybrid approach and to bring it into the causal framework, the output data from the RF classifier with the selected variables were then presented to the gPath to fit a causal model to the data. There were six steps in each path modeling, including model specification, model identification, model estimation, model testing, model modification, and model validation. Model specification involves detecting relationships among a set of study variables. In this step, a graphical presentation of the model is applied to create a conceptual model. Model identification includes formulating the relationships presented in the model specification phase and guaranteeing that the model is fitted properly. In the model estimation step, the set of equations is solved simultaneously to estimate the model fitting parameters.^20^ In this study, the weighted least square mean and variance-adjusted estimator was employed, which is a robust one and does not assume a normal distribution for variables. In addition, it provides the best option for modeling categorical or ordered data. The indices, including chi-square test/degree of freedom values ( $\frac{\chi^{2}}{df}$ ) below five, Tucker-Lewis index (TLI), and comparative fit index (CFI) values over 0.90 were used for model goodness of fit.^21,22^ The root means square error of approximation (RMSEA) was the next measure of goodness-of-fit, with values below 0.05 being considered a good fit and values up to.08 representing acceptable errors in the population.^20^ For an inadequate model, the model modification includes adjusting an identified and estimated model through modification indices provided by the model. In this study, the bootstrap method was utilized for model validation.

## Results

 From March 2015 to March 2016, IRTIRS registered 384 614 traffic crashes. The suburban area crashes comprised 52 524 (13.66%) of the causalities. The fatality rate among these crashes was 4.6% (2399 cases). Table 1 provides details about the frequency distribution of crash scenes, vehicles, and driver-related variables describing the crashes.

###  Results of the random forests model

**Figure 1:**
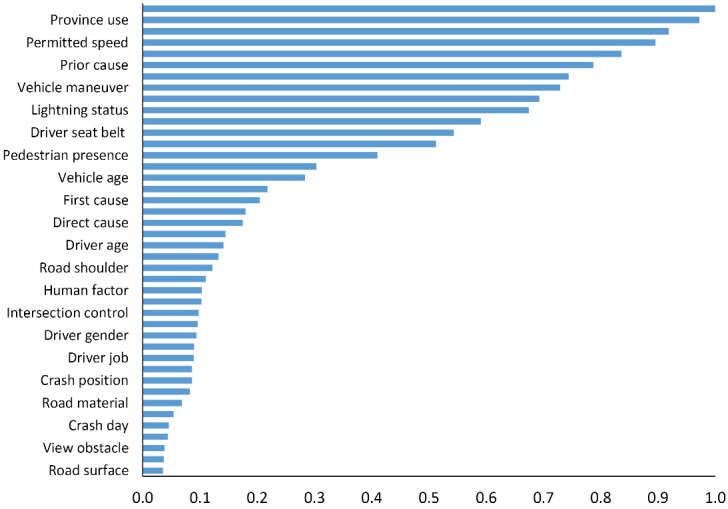
Variables’ importance based on the random forest model

 The results of RF feature selection demonstrated that 12 variables, namely, collision type, distinct, driver misconduct, permitted speed, driver’s license, plaque description, vehicle maneuver, vehicle type, lighting status, passenger presence, driver seat belt, and land use, were derived as significant variables. Risk estimates and corresponding standard errors were 0.046 and 0.001 for the training and test sets. Figure 1 recapitulates the results of the RF model in more detail.

###  Results of the hybrid RF- gPath model

**Figure 2:**
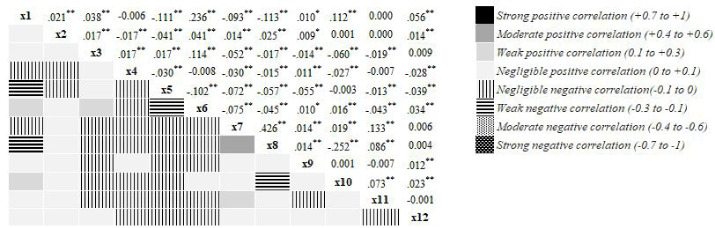
Correlation matrix for variables within the hybrid RF-gPath model. *Note*. RF: Random forests. ^**^Correlation is significant at the 0.01 level (2-tailed). ^*^ Correlation is significant at the 0.05 level (2-tailed). x1: Passenger presence, x2: Lighting status, x3: Land use, x4: Collision type, x5: Distinct; x6: Permitted speed; x7: Vehicle type, x8: Plaque description, x9: Vehicle maneuver, x10: Driver license, x11: Driver seat belt, and x12: Driver misconduct

 Although the RF method was used to select variables, understanding the potential for multicollinearity between the inventory variables, we checked the correlation between independent variables to ensure they were not highly correlated. Figure 2 shows a correlation matrix for all the variables introduced to the causality model. The color coding represents how correlated two variables are, with dark blue and dark red squares representing a strong positive correlation ( + 0.7 to + 1) and a strong negative correlation (-1 to -0.7), respectively.^23^ According to the figure, the correlations between variables are not strong enough for any substantial collinearity or multicollinearity.^24^

**Figure 3:**
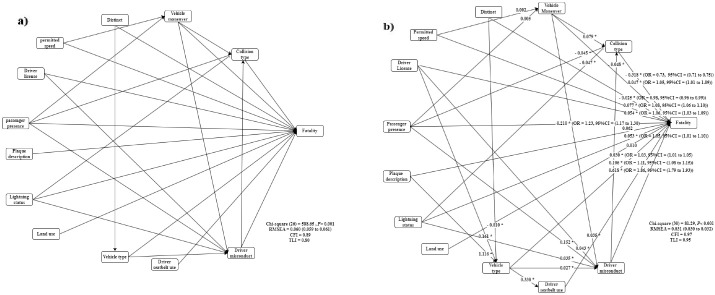
**(a)** Hypothesized conceptual model and **(b)** perfect fitted model with standardized path coefficients in assessing the effects of risk factors on fatality in suburban area crashes (^*^P < 0.05)

 A conceptual model of variables extracted from the RF model (Figure 3a) was constructed to answer the research question. Figure 3b illustrates the modified model, where the values on the arrows represent standardized regression coefficients from one variable to another, which are the direct effects. The modified model fitted the data reasonably enough with $\chi_{30}^{2}$ = 81.29, *P* < 0.001, $\chi^{2}/df$ = 2.71 < 5, CFI = 0.97 > .9, TLI = 0.95 > 0.9, and RMSEA = 0.031 < 0.08 (90% confidence interval [CI]: 0.030 to 0.032). Table 2 provides direct, indirect, and total effects ending in the outcome. Bootstrap confirmed the model validation as having an acceptable overlap of method confidence intervals with model-derived confidence intervals and negligible biases.

**Table 2:** Standardized direct, indirect, and total effects ending in the outcome (fatality)

**Variables/Effect**	**Estimate**	**SE**	***P*** ** value**
Collision type			
Direct	-0.318	0.012	0.001
Indirect	-0.108	0.041	0.001
Total	-0.426	0.016	0.001
Maneuver			
Direct	0.047	0.022	0.029
Indirect	0.000	0.000	0.001
Total	0.047	0.022	0.029
Distinct			
Direct	-0.025	0.008	0.002
Indirect	0.000	0.000	0.001
Total	-0.025	0.008	0.002
Permitted speed			
Direct	0.077	0.008	0.001
Indirect	0.000	0.000	0.001
Total	0.077	0.008	0.001
Driver license			
Direct	0.054	0.015	0.001
Indirect	0.023	0.005	0.001
Total	0.077	0.015	0.001
Passenger presence			
Direct	0.210	0.026	0.001
Indirect	0.104	0.010	0.001
Total	0.314	0.027	0.001
Plaque description			
Direct	0.062	0.041	0.105
Indirect	0.084	0.030	0.005
Total	0.146	0.027	0.001
Lightning status			
Direct	0.053	0.020	0.008
Indirect	0.000	0.000	0.001
Total	0.053	0.020	0.008
Land use			
Direct	0.010	0.021	0.621
Indirect	0.000	0.000	0.001
Total	0.010	0.021	0.621
Vehicle type			
Direct	0.030	0.010	0.027
Indirect	0.049	0.005	0.005
Total	0.079	0.027	0.005
Driver seatbelt usage status			
Direct	0.106	0.016	0.001
Indirect	0.000	0.000	0.001
Total	0.106	0.016	0.001
Driver misconduct			
Direct	0.618	0.019	0.001
Indirect	0.000	0.000	0.001
Total	0.618	0.019	0.001

*Note*. SE: Standard error.

###  Indirect effects

 All coefficients on the perfect fitted model were statistically significant at the 0.05 level of significance, except for the path from the vehicle plaque description and land use toward fatality, as well as the path from permitted speed and the presence of passenger toward vehicle maneuver. The results revealed that the presence of a passenger increased the odds of fatal crashes by 1.23 times (1.23, 1.17 to 1.30). Drivers not wearing a seatbelt had 11% higher odds of dying in a suburban area crash (1.11, 1.08 to 1.15). The odds of fatality increased by 1.86 for drivers engaging in misconduct (1.86, 1.79 to 1.93).

 Furthermore, the findings demonstrated a significant direct relationship between fatality vehicle maneuver (odds ratio [OR] = 1.05, 95% CI: 1.01 to 1.09), permitted speed (OR = 1.08, 95% CI: 1.06 to 1.10), driver license (OR = 1.06, 95% CI: 1.03 to 1.09), lightning status (1.05, 1.01 to 1.10), and vehicle type (1.03, 1.01 to 1.05). Further, a significant converse relationship was found between fatality and collision type (0.73, 0.71 to 0.75), as well as fatality and distinct (0.98, 0.96 to 0.99).

###  Mediation effects of risk factors on fatality outcome

 The mediated path model indicated that collision type mediated the effect of vehicle maneuver (*β* = 0.079,95% CI = 0.059 to 0.099), presence of passenger (*β* = -0.045,95% CI = -0.070 to -0.020), lightening status (*β* = -0.047,95% CI = -0.063 to -0.031), and driver misconduct (*β* = -0.045,95% CI = -0.058 to -0.038) on fatality. The strongest association was observed between fatality and vehicle maneuver. Although the paths from permitted speed and the presence of passengers with fatality were mediated by vehicle maneuvers, they were insignificant. Vehicle type mediated the effect of distinct (*β* = -0.010,95% CI = -0.012 to - 0.008), driver license (*β* = 0.161,95% CI = 0.155 to 0.167), and plaque description (*β* = 1.116,95% CI = 1.106 to 1.126) on fatality.

 There was a positive indirect effect for vehicle type on fatality through the driver seat belt (*β* = 0.338,95% CI = 0.320 to 0.356). Furthermore, vehicle maneuver (*β* = 0.035,95% CI = 0.015 to 0.055), driver license (*β* = 0.043,95% CI = 0.031 to 0.055), presence of a passenger (*β* = 0.152,95% CI = 0.123 to 0.181), lightning status (*β* = 0.035,95% CI = 0.017 to 0.053), and vehicle type (*β* = 0.027,95% CI = 0.011 to 0.043) were significantly and indirectly related to fatality through driver misconduct (Figure 3b).

## Discussion

 This is the first study that discovered the applicability of the innovative HRF-gPath model for detecting causal relationships and predicting fatality in suburban crashes. The proposed novel HRF-gPath chose a reasonable number of features and showed their direct and indirect relationships.

 Interestingly, the association between vehicle maneuver, presence of passenger, lightning status, and driver misconduct paths with fatality were mediated by collision type. Moreover, distinct, driver’s license and plaque descriptions affected the vehicle type and, consequently, fatality, which is consistent with the findings of a previous study.^25^ The relationship between vehicle types by fatality was mediated by seat belt use. Furthermore, driver misconduct played a mediator role in assessing the relationship between fatality and variables such as vehicle maneuver, driver license, presence of a passenger, lightning status, and vehicle type. Collision type, vehicle type, seat belt use, and driver misconduct demonstrated a significant relationship with fatality. Therefore, this explored model could be considered a typical practical, theoretical framework to explain how the collision type, vehicle type, seat belt use, and driver misconduct can predict and mediate fatality in suburban crashes. Further studies can modify and establish this model.

 Based on the results of the present study, vehicle maneuver, presence of a passenger, lightning status, and driver misconduct could be considered significant predictors of collision type. The significant relation between vehicle maneuvers and collision type indicates that different vehicle maneuvers would lead to different collision types. Overtaking while driving, as the main cause of head-on collisions with serious consequences, can be a salient example of this relationship, as reported in other studies.^26,27^ Consistent with the results of international research, the presence of a passenger may reduce attention to the driving task and exert direct or indirect psychological pressure to drive less safely. In the same vein, it can be assumed that the presence of a passenger may lead to increased stress and thus reduced driving performance.^28^ However, we cannot make any assumptions about the risky role of passenger presence, which is similar to the finding of the study conducted by Orsi et al. They concluded that young drivers, carrying passengers, were particularly vulnerable in single-vehicle collisions; yet, for adult drivers, this collision was more harmful if the driver was alone in the vehicle,^16^ which is in line with the results regarding the relationship between lightning status and collision type. The studies assessing rear-end crash exposure methodology revealed that daytime was attributed to many rear-end collisions.^29^ Studies have reported driver misconduct as a predictor of collision type. Goel and Sachdeva had studied the reasons for the collisions, their kind when they occurred, and the kind of the involved vehicle. They found that head-on or rear-end collisions are mainly due to driver misconduct.^30^

 Considering the division of distinct (tourism destination, capital city destination, and free zone), the relationship between the distinct and the vehicle type is quite clear. Based on the results of this study, the distribution of heavy vehicles in the capital destination has a different pattern than in a tourist destination and the free zone. Tehran, the capital of Iran, is the economic center of Iran, with more than 45% of large industrial factories.^31^ Therefore, these factories increase the use of heavy vehicles for road freight transport. Similar studies represented that freight vehicles are heavier and increase the kinetic energy in accidents compared to passenger vehicles. In addition, capital cities usually have limited infrastructure for freight infrastructure, including loading space, road space, and parking, to accommodate the increasing freight traffic. These limitations further challenge the safe and efficient operation of heavy vehicles.^32^

 According to the results of similar studies in Iran, car by itself has effects whether or not drivers decide to use seat belts. For example, sport utility vehicles and van drivers are less likely to use seat belts.^33,34^

 Among all variables, the presence of a passenger was a stronger predictor of diver misconduct. Talking to the passenger has been identified as a distractor and a predictor of driver misconduct.^35^ It has been concluded that professional drivers have a lower probability of risky driving behaviors. However, this is in contrast with the findings of a study by Mekonnen et al, indicating that diver misconduct is common among professional drivers.^36^

 As a third significant predictor of driver misconduct, vehicle maneuver plays a crucial role. Based on the findings of similar studies, the likelihood of misconduct increases by 2.98 and 2.15 times for drivers who engage in overspeeding and those who frequently make dangerous overtakes, respectively.^37^ Lightning status is the other significant predictor of driver misconduct. There is solid evidence from some studies that driving in dim light makes it harder to prevent crashes. As the number of miles traveled at night is significantly lower than during the day, drivers are more likely to drive faster during the daytime than at night.^37,38^ In terms of the relationship between driver misconduct and vehicle type, it is believed that as the key participant in the goods industry, drivers of heavy vehicles are one of the main factors of traffic safety. In the study of traffic collisions involving heavy vehicles, it was declared that 90% were found to be the result of driver misconduct.^39^

 As the first limitation, there is no precise and detailed registry system in the country to combine this information with hospital information. As a result, only information on death at the scene is available, and therefore the results cannot be generalized to cases of death in the hospital. Another problem of this study is that accidents are probably not reported fully to the authorities. Focusing on the data between 2015 and 2016 and a restriction to access data from 2016 to 2021, which would enlarge and improve this research, can be considered the main limitation of this study. Like most classification problems, this study is limited by its imbalanced data. Although balancing data before conducting a random forest model can improve model performance and accurate evaluation metrics, it may lead to information loss, time and computational resources increase, and real-world imbalance mismatch. Hence, experimenting with both balanced and imbalanced datasets to assess the impact on model performance and choose the approach that best aligns with the problem is recommended for further studies.

 On the other hand, this study was based on information from six densely populated provinces of the country, thus this can be considered the study’s first strength, making the results generalizable. This study introduced a hybrid approach for analyzing traffic crash data to develop a parsimonious model for suburban area crashes, which can be another study strength.

**Highlights**

- We proposed a hybrid random forest generalized path analysis (HRF-gPath) model.
- Collision, vehicle type, seatbelt, and misconduct predict and mediate crash events.
- The HRF-gPath model provides a good fit for identifying suburban crash risk factors.

## Conclusion

 The proposed novel HRF-gPath model helped us identify reasoned pathways of fatal crashes in suburban areas. When exogenous and mediator variables are modeled together, all may predict fatality. As mediator variables, collision type, vehicle type, seat belt use, and driver misconduct originate from risk factors underlying this predicament. It is suggested that further research explores the unseen biases of the issue. Healthcare providers, police, and psychologist should consider the dominance of mediators explored in this study while designing prevention programs for suburban area crashes.

## Acknowledgments

 We are thankful to all people who helped us to conduct this study. The authors would like to acknowledge the staff of the Road Traffic Injury Research Center of Tabriz University of Medical Sciences for supporting this study. This is a database report from a Ph.D. thesis registered in Tabriz University of Medical Sciences with No. 64041 by Fatemeh Jahanjoo.

## Authors’ Contribution

**Conceptualization:** Mohammad Asghari-Jafarabadi, Homayoun Sadeghi-Bazargani, Fatemeh Jahanjoo, Seyyed Teymoor Hosseini.

**Data curation: **Mohammad Asghari-Jafarabadi, Homayoun Sadeghi-Bazargani, Fatemeh Jahanjoo.

**Formal analysis: **Mohammad Asghari-Jafarabadi, Fatemeh Jahanjoo

**Funding acquisition:** Mohammad Asghari-Jafarabadi, Homayoun Sadeghi-Bazargani.

**Investigation:** Homayoun Sadeghi-Bazargani, Mohammad Asghari-Jafarabadi.

**Methodology: ** Mohammad Asghari-Jafarabadi, Homayoun Sadeghi-Bazargani, Fatemeh Jahanjoo.

**Project administration: **Mohammad Asghari-Jafarabadi.

**Resources: **Mohammad Asghari-Jafarabadi, Homayoun Sadeghi-Bazargani.

**Software:** Mohammad Asghari-Jafarabadi, Fatemeh Jahanjoo.

**Supervision: **Mohammad Asghari-Jafarabadi.

**Validation:** Mohammad Asghari-Jafarabadi, Homayoun Sadeghi-Bazargani, Mohammad Ali Mansournia.

**Visualization: **Mohammad Asghari-Jafarabadi, Fatemeh Jahanjoo.

**Writing–original draft: **Mohammad Asghari-Jafarabadi, Fatemeh Jahanjoo.

**Writing–review & editing: **Mohammad Asghari-Jafarabadi, Homayoun Sadeghi-Bazargani, Fatemeh Jahanjoo, Mohammad Ali Mansournia, Seyyed Teymoor Hosseini.

## Competing Interests

 The authors declare no conflict of interests associated with this manuscript.

## Ethical Approval

 This study was approved by Institutional Review Board of Tabriz University of Medical Sciences (TUOMS) with ethics code: IR.TBZMED.REC.1398.1244.

## Funding

 This study was based on data from Fatemeh Jahanjoo’s Ph.D. thesis, which was financially supported by the Research Deputy of the Tabriz University of Medical Sciences (TUOMS) under Grant No. 64041.
